# Differential Radiosensitivity Phenotypes of DNA-PKcs Mutations Affecting NHEJ and HRR Systems following Irradiation with Gamma-Rays or Very Low Fluences of Alpha Particles

**DOI:** 10.1371/journal.pone.0093579

**Published:** 2014-04-08

**Authors:** Yu-Fen Lin, Hatsumi Nagasawa, John B. Little, Takamitsu A. Kato, Hung-Ying Shih, Xian-Jin Xie, Paul F. Wilson Jr., John R. Brogan, Akihiro Kurimasa, David J. Chen, Joel S. Bedford, Benjamin P. C. Chen

**Affiliations:** 1 Department of Radiation Oncology, University of Texas Southwestern Medical Center at Dallas, Dallas, Texas, United States of America; 2 Department of Environmental and Radiological Health Sciences, Colorado State University, Fort Collins, Colorado, United States of America; 3 Department of Genetics and Complex Diseases, Harvard School of Public Health, Boston, Massachusetts, United States of America; 4 Department of Clinical Sciences, University of Texas Southwestern Medical Center at Dallas, Dallas, Texas, United States of America; 5 Department of Biosciences, Brookhaven National Laboratory, Upton, New York, United States of America; 6 Institute of Regenerative Medicine and Biofunction, Graduate School of Medical Science, Tottori University, Tottori, Japan; University of California Davis, United States of America

## Abstract

We have examined cell-cycle dependence of chromosomal aberration induction and cell killing after high or low dose-rate γ irradiation in cells bearing DNA-PKcs mutations in the S2056 cluster, the T2609 cluster, or the kinase domain. We also compared sister chromatid exchanges (SCE) production by very low fluences of α-particles in DNA-PKcs mutant cells, and in homologous recombination repair (HRR) mutant cells including Rad51C, Rad51D, and Fancg/xrcc9. Generally, chromosomal aberrations and cell killing by γ-rays were similarly affected by mutations in DNA-PKcs, and these mutant cells were more sensitive in G_1_ than in S/G_2_ phase. In G_1_-irradiated DNA-PKcs mutant cells, both chromosome- and chromatid-type breaks and exchanges were in excess than wild-type cells. For cells irradiated in late S/G_2_ phase, mutant cells showed very high yields of chromatid breaks compared to wild-type cells. Few exchanges were seen in DNA-PKcs-null, Ku80-null, or DNA-PKcs kinase dead mutants, but exchanges in excess were detected in the S2506 or T2609 cluster mutants. SCE induction by very low doses of α-particles is resulted from bystander effects in cells not traversed by α-particles. SCE seen in wild-type cells was completely abolished in Rad51C- or Rad51D-deficient cells, but near normal in Fancg/xrcc9 cells. In marked contrast, very high levels of SCEs were observed in DNA-PKcs-null, DNA-PKcs kinase-dead and Ku80-null mutants. SCE induction was also abolished in T2609 cluster mutant cells, but was only slightly reduced in the S2056 cluster mutant cells. Since both non-homologous end-joining (NHEJ) and HRR systems utilize initial DNA lesions as a substrate, these results suggest the possibility of a competitive interference phenomenon operating between NHEJ and at least the Rad51C/D components of HRR; the level of interaction between damaged DNA and a particular DNA-PK component may determine the level of interaction of such DNA with a relevant HRR component.

## Introduction

The catalytic subunit of DNA dependent protein kinase (DNA-PKcs) is the key regulator of non-homologous end-joining (NHEJ), the predominant DNA double-strand break (DSB) repair mechanism in mammals. DNA-PKcs is recruited to DSBs through the DNA-binding heterodimer Ku70/80, and together with these factors form the kinase active DNA-PK holoenzyme [1]. The biological significance of DNA-PKcs first became evident with the finding that mutation within the gene encoding DNA-PKcs led to severe combined immunodeficiency (SCID) in mice and other animals [2],[3]. The other major phenotypic trait coffered by DNA-PKcs mutations was severe hypersensitivity to ionizing radiation (IR) and radiomimetic chemicals [4]. Kurimasa et al. confirmed the requirement of DNA-PKcs kinase activity for DSB rejoining after irradiation [5]. DNA-PKcs activation upon IR or treatment with radiomimetic chemicals rapidly results in phosphorylation of DNA-PKcs in the S2056 and the T2069 phosphorylation cluster regions [6]–[9]. Studies of DNA-PKcs mutant cell lines indicate that these phosphorylations are required for full DSB repair capacity and normal cellular radiosensitivity.

DNA-PKcs and its downstream NHEJ components are active in all cell cycle phases. In contrast, homologous recombination repair (HRR), another major DSB repair mechanism, contributes to DSB repair and cellular survival only during S and G_2_ phases [10], [11]. To clarify the significance of DNA-PKcs activities in NHEJ-mediated DSB repair and in radiosensitivity, it is important to study synchronous cell populations at different phases throughout the cell cycle. We reported previously that cells expressing DNA-PKcs with mutations in the S2056 cluster, the T2609 cluster, or the PI3K kinase domain have clear differences in radiosensitivities when mutant cells were irradiated in the G_1_ phase [12]. Expression of DNA-PKcs with mutations in the T2609 cluster (L-3) or in the PI3K kinase domain (L-8, L-9, L-10, and L-11) results in extreme radiosensitivity, similar to that of Ku70/80-deficient xrs-5 and xrs-6 cells; however, mutations in the S2056 cluster (L-12) result in intermediate radiosensitivity [12].

DNA-PKcs mutants, V3 (DNA-PKcs null) and irs-20 (extreme c-terminal motif mutant) cause extreme and moderate radiosensitivity, respectively. These radiosensitive mutant cell strains respond to radiation in a cell-cycle-dependent manner and display enhanced radiation-induced cell cycle delay. In plateau phase G_1_ cells, a greatly reduced potentially lethal damage repair (PLDR), sub-lethal damage repair (SLDR), and a great reduction or absence of a dose-rate effect are observed [13]–[17]. Chinese Hamster Ku70/80-deficient xrs-5 and xrs-6 cell lines are more radiosensitive than wild-type cells and the radiosensitivity does not depend on cell cycle stage. In addition, these cells show no PLDR and no dose-rate effect. In these respects xrs-5 and xrs-6 are similar to ATM-deficient cell strains [18]–[25].

In connection with the DNA-PKcs phosphorylation-defective mutants described above, we have also reported other results indicating that HRR was required for the induction of SCEs by alpha particles [26], [27]. We have further investigated this in more detail in the present study by comparing SCE induction after very low doses of α-particles in cells that express the mutations in DNA-PKcs described above. The doses were sufficiently low that the observed levels of induced SCE could be attributed to effects produced in unirradiated “bystander” cells. In the present study, we compared radiosensitivity phenotypes among cell lines that express mutant versions of components of NHEJ system (DNA-PKcs, Ku80) or components of HRR system. We also examined the cell cycle dependence of chromosomal aberration induction and cell killing after high and low dose-rate γ irradiation.

## Materials and Methods

### Cell lines and synchrony

For these studies, we employed the wild-type Chinese hamster cell lines CHO [28] and AA8 [29], NHEJ- deficient mutant lines xrs-5 [30] and V3 [31], HRR mutant lines irs-3 [32], CL-V4B [33], 51D1 [34], Fanconi anemia (FA) mutant (KO40) [35], and cell lines derived from DNA-PKcs-null V3 cells complemented with human DNA-PKcs cDNA containing amino acid substitutions at various positions [5]–[7], [12], [36] that are described in Tables 1 and 2. The cells were maintained at 37°C in a humidified 95% air/5% CO_2_ atmosphere in Eagle's minimal essential medium (MEM) supplemented with 10% heat-inactivated fetal bovine serum (FBS), penicillin (50 mg/ml), and streptomycin (50 µg/ml). When the cultures approached 30% confluence in T-25 tissue culture flasks or Mylar-dishes, the normal growth medium was replaced twice at 24-hour intervals with isoleucine-deficient MEM containing 5% 3× dialyzed FBS to synchronize the cells in the G_1_ phase [37]. G_1_ synchronized cells were released in normal growth medium for 12 hours to achieve synchrony in late S/G_2_ phase of cell cycle. As shown in Table 2, G_1_ cell populations were relatively pure as judged by quantification of bromo-2′-deoxyuridine (BrdU) following a 30 minute-pulse labeling; BrdU specifically labels S phase cells. Experiments shown were performed with G_1_ populations containing 1–12% S phase cells.

**Table 1:** Amino acid substitutions in the DNA-PKcs constructs.

Cell line/code	DNA-PKcs mutants	S2056 cluster^1^					T2609 cluster^1^						PI3K		Rad-Sens^4^
		S2023	S2029	S2041	S2051	S2056	T2609	S2612	T2620	S2624	T2638	T2647	Y3715	D3921	
L-1	wild type														N*
L-3	V3-6A (T2609 cluster to A)						A	A	A	A	A	A			SSS*
L-6	V3-T2609A						A								S
L-7	V3 (empty vector)														SSS*
L-10	V3-KC23 (kinase dead)^2^												D		SSS*
L-11	V3-KD51 (kinase dead)^3^													N	SSS*
L-12	V3-5A (S2056 cluster to A)	A	A	A	A	A									S*
L-14	V3-3A (T2609A/T2638A/T2647A)						A				A	A			SS

1 . Serine (S) and threonine (T) were replaced with alanine (A) at S2056 and T2609 cluster sites.

2 . V3-KC23 mutant carries a frame-shift at position of amino acid 3715 that results in truncation of the protein after 10 amino acids and loss of the entire PI3K kinase domain.

3 . In V3-KD51 mutant aspartic acid (D) at 3921 is replaced with asparagine (N).

4 . N: Normal; S: Slightly sensitive; SS, Sensitive; SSS: Very sensitive. Levels are from a previous report on survival responses from this laboratory; Sensitivities marked by the * were those confirmed independently in the present study.

**Table 2:** Induction of SCE with 0.7α-particle irradiation.

Cell line	Defective gene	Origin	MDT^1^ (hrs)	% in S-phase	SCE per chromosome	
					0 mGy	0.7 mGy
Wild type						
CHO	None	Wild type	14	3.4	0.336±0.030	0.437±0.058
AA8	None	CHO	13	1.4	0.332±0.009	0.440±0.016
NHEJ mutants						
xrs-5	Ku80−/−	CHO	18	10.5	0.442±0.048	1.281±0.170^2^
V3	DNA-PKcs−/−	AA8	18	0.8	0.387±0.069	0.607±0.073^3^
HR/FA mutants						
Irs-3	Rad51C	V79	14	6.3	0.161±0.009	0.169±0.001
CL-V48	Rad51C	V79	15	3.4	0.156±0.008	0.148±0.006
51D1	Rad51D	AA8	24	7.9	0.326±0.015	0.302±0.026
KO40	Fancg/xrrc9	AA8	20	13.9	0.332±0.014	0.443±0.017^3^
DNA-PKcs mutants						
L-1	V3 (wild-type)	V3	18	0.8	0.303±0.098	0.430±0.110
L-3	V3-6A (T2609 cluster to A)	V3	14	7.7	0.347±0.013	0.357±0.028
L-6	V3-T2609A	V3	17	12.2	0.305±0.069	0.327±0.003
L-10	V3-KC23 (kinase dead)	V3	18	12.1	0.421±0.042	1.316±0.014
L-11	V3-KD51 (kinase dead)	V3	16	2	0.505±0.056	0.916±0.002
L-12	V3-5A (S2056 cluster to A)	V3	16	2	0.269±0.012	0.425±0.053
L-14	V3-3A (T2609A/T2638A/T2647A)	V3	ND	6.1	0.332±0.014	0.332±0.014

1 . MDT: mean doubling time.

2 . Cells were irradiated with 0.35 mGy.

3 . Cells were irradiated with 0.18 mGy;

### Irradiation and colony formation

For acute high dose-rate exposures, cells were irradiated with a J. L. Shepherd and Associates irradiator that emitted ^137^Cs γ-rays at a dose rate of 2.5 Gy/min. For the low dose rate ^137^Cs γ-ray irradiations, a J. L. Shepherd and Associates Model 81-14 beam irradiator containing a single relatively low activity (nominal 28 Ci) ^137^Cs source placed 100 cm above a water-jacketed CO_2_ incubator that was maintained at 37°C. For the colony formation assay, different dose rates were achieved by placing cultures on shelves in the incubator located at different distances below the source during 8 days of continuous low-dose-rate irradiation [17].

For α-particle irradiation, cells were cultured on the Mylar dishes and were placed over a Mylar window in the exposure well of specially constructed irradiator that provides a uniform source of well-characterized 3.07 MeV α-particles. The source consisted of 296 MBq of ^238^PuO_2_ electrodeposited onto one side of a 100-mm diameter stainless steel disk. The cells were irradiated from below in a helium environment, and the α-particles traversed a reciprocating collimator before reaching the Mylar window. The target-to-source distance is 42 mm in helium gas, 6 mm in air, and 3 mm in Mylar. Dose was controlled by a timer and precision photographic shutter, which allowed accurate doses of irradiation [38].

Survival curves were obtained by measuring the colony-forming ability of irradiated cell populations. Cells were plated immediately after irradiation onto 100-mm plastic Petri dishes and incubated for 8–10 days. Colonies were fixed with 100% ethanol and stained with 0.1% crystal violet solution. A colony with more than 50 cells was scored as a survivor.

### Chromosome analysis

Synchronized G_1_-phase cells were subcultured into three T25 plastic flasks and cultured in fresh medium containing 10 µM BrdU for the first and second cycle cells after irradiation. At 4 hour intervals beginning 13 hours after subculture, colcemid was added to one of the three flasks to arrest cells in the first mitosis after subculture; total sampling time thus covered 12 hours. Most cells moved into the first and second rounds of mitosis after 13–18 hours and 25–35 hours post irradiation, respectively. The cells were fixed in methanol∶acetic acid (3∶1), and chromosomes were spread by air dry method [39]. The differential staining of cells in first and second rounds of mitosis were performed by the fluorescence plus Giemsa technique [40]. Chromosome aberration was analyzed at peak mitotic indices after irradiation. In brief, exponentially growing cells were irradiated with ^137^Cs γ-rays at a dose rate of 2.5 Gy/min. Colcemid was added to a final concentration of 0.1 µg/ml at 30 min after irradiation, and the cells were harvested 4 hours later; under these conditions the mitotic cells collected would have been in late S/G_2_ phase of cell cycle at the time of irradiation [41]. For sister chromatid exchange (SCE), irradiated cells were cultured in complete MEM containing 10 µM BrdU for two rounds of cell replication, and colcemid was added prior to the peak of second mitoses [42].

### Statistical analysis

All statistical analyses were performed using Prism GraphPad (version 6.02) software. Statistical significance was diagnosed by *t*-test and defined as p<0.05, but values of p<0.01, p<0.001 and p<0.0001 are shown as well to indicate level of confidence.

## Results

**Figure 1:**
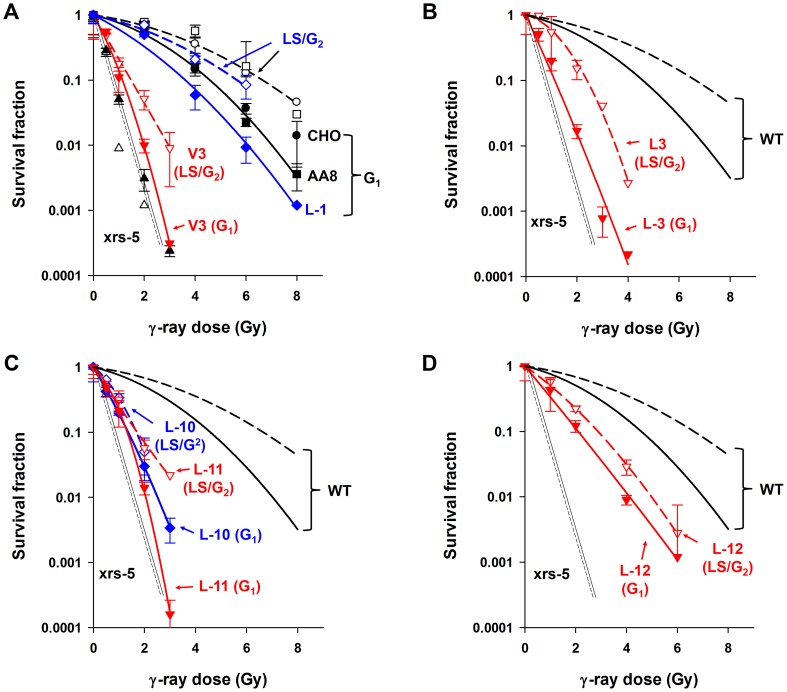
Effect of radiosensitivities on G_1_- and late S/G_2_-phase cells. G_1_ (closed symbols) and late S/G_2_ (LS/G_2_, open symbols) synchronized cells [CHO and AA8 (WT), xrs-5, (A) V3, L-1, (B) L-3, (C) L-10, L-11, and (D) L-12] were irradiated by γ–rays and were reseeded immediately for analysis of colony formation. A colony with more than 50 cells was scored as a survivor. The results are means ± SEMs from more than three independent experiments with each cell line.

Our previous investigation with G_1_-synchronized V3 cells and derivative cell lines expressing DNA-PKcs mutants revealed the contributions of the S2056 and T2609 clusters and the PI3K domain to NHEJ-dependent DSB repair and clonogenic survival [12]. In the current study, we examined the impact of DNA-PKcs domains on radiosensitivity for cell killing and chromosomal aberration induction following irradiation during different cell cycle phases. Radiosensitivities for cell killing of wild-type and various NHEJ-deficient mutant cells (Table 1) were investigated in synchronous cell populations in G_1_ or late S/G_2_ phases of the cell cycle. The synchronized cell populations were irradiated with γ-rays and reseeded to evaluate clonogenic survival (Fig. 1). In comparison to the wild-type Chinese Hamster cells (CHO and AA8), Ku70/80-deficient xrs-5 cells were extremely radiosensitive, and, as previously reported, survival was not affected by the stage of the cell cycle at the time of irradiation [24]. DNA-PKcs-deficient V3 cells were also highly radiosensitive, but cells irradiated in the late S/G_2_ phase of cell cycle had enhanced survival compared to those irradiated in G_1_ (Fig. 1A). Similarly, cells expressing DNA-PKcs mutants defective at the T2609 cluster (L-3, Fig. 1B), the PI3K kinase domain (L-10 and L-11, Fig. 1C), or the S2056 cluster (L-12, Fig. 1D) all exhibited higher cell survival when irradiated in late S/G_2_ than in G_1_, although this effect was not as prominent as that observed for the wild-type CHO cells (Table 3).

**Table 3:** Effect of cell cycle on radiosensitivity.

Cell line	D10 dose (Gy)^1^		Ratio of sensitivities of S/G_2_ to G_1_ cells^2^
	G_1_ phase	S/G_2_ phase	
CHO	4.1	6.4	1.6
AA8	4.6	7.7	1.7
xrs-5	0.8	0.8	1
V3	0.9	1.2	1.3
L-3	0.9	1.3	1.4
L-10	0.9	1.3	1.4
L-11	0.7	1.2	1.7
L-12	2.2	3.3	1.5

1 . D10, radiation dose required to reduce survival to 10% in G_1_ or S/G_2_ synchronized cells.

2 . Calculated by dividing D10 of S/G_2_ phase by D10 of G_1_ phase.

**Figure 2:**
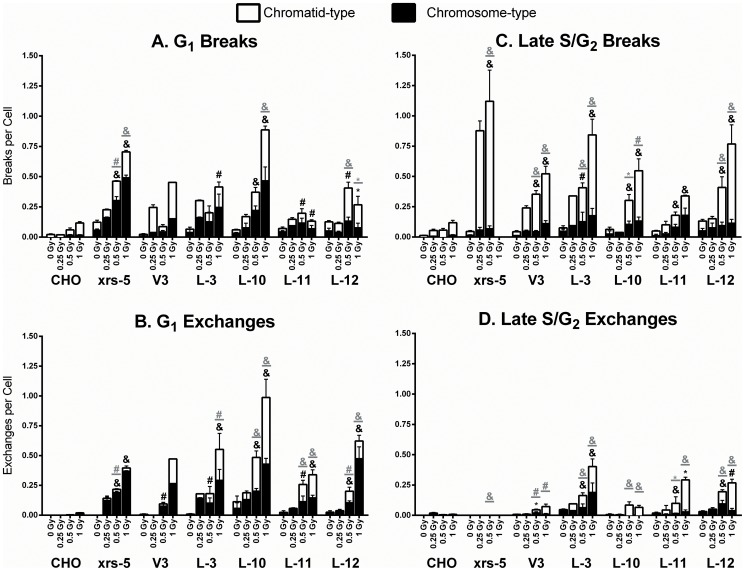
Gamma-ray induced chromosomal aberrations in G_1_ and S/G_2_ phases. Wild-type (CHO), NHEJ-deficient mutant lines (xrs-5, V3), and DNA-PKcs mutant strains were irradiated with doses of 0, 0.25, 0.5 or 1.0 Gy γ–rays during the G_1_ or late S/G_2_ phases of cell cycle. Chromatid-type (□) and chromosome-type (▪) aberrations were analyzed and scored as breaks (A, C) or exchanges (B, D). The results are means ± SEMs from more than three independent experiments. Statistical analyses on the 0.5 Gy or 1 Gy-induced aberrations relative to CHO cells were performed using *t*-test. *, p<0.05; #, p<0.01; &, p<0.001; the black and gray symbols indicate the significant differences in chromosome-type and chromatid-type aberrations, respectively.

Wild-type, xrs-5 and DNA-PKcs mutant cells were examined for chromosome and chromatid-type aberrations induced by various doses of γ-rays (Fig. 2). Generally, DNA-PKcs mutant cells displayed more chromosomal aberrations per unit dose than wild-type CHO cells regardless whether irradiation occurred at G_1_ or late S/G_2_ phases. When the cells were irradiated in the G_1_ phase, relative to wild-type cells significantly elevated frequencies of radiation-induced breaks and exchanges were observed; levels of these aberrations were similar in cells with DNA-PKcs protein defective at the T2609 cluster, the PI3K domain or the S2056 cluster and in Ku-deficient xrs-5 cells (Fig. 2A, 2B). As previously noted, relatively few chromatid-type aberrations were induced by G_1_ irradiation of wild-type cells but were numerous in NHEJ-deficient mutant cells (Fig. 2A, 2B) [12], [15]. When the cells were irradiated in the late S/G_2_ phase of the cell cycle, frequencies of radiation-induced chromosome breaks or deletions were similar in all DNA-PKcs mutant cells and were significantly different from wild-type cells (Fig. 2C). There were obvious differences between xrs-5 cells and DNA-PKcs mutant cells: After irradiation with 0.5 Gy in late S/G2 phase, approximately 3 to 5 times more chromatid breaks were observed in xrs-5 cells than in DNA-PKcs mutant cells (Fig. 2C). It is notable that after 1 Gy irradiation during the late S/G_2_ phase of the cell cycle, very few xrs-5 mitotic cells were collected due to a long delay at the G_2_ checkpoint as previously reported [43]. Results indicated that perhaps DNA-PKcs mutant cells showed lower frequencies of chromatid type exchanges (triradials and quadriradials) when the cells were irradiated in the late S/G_2_ phase than when cells were irradiated during the G_1_ phase (Figs. 2B and 2D); L-3 cells were an exception to this, as discussed further below.

**Figure 3:**
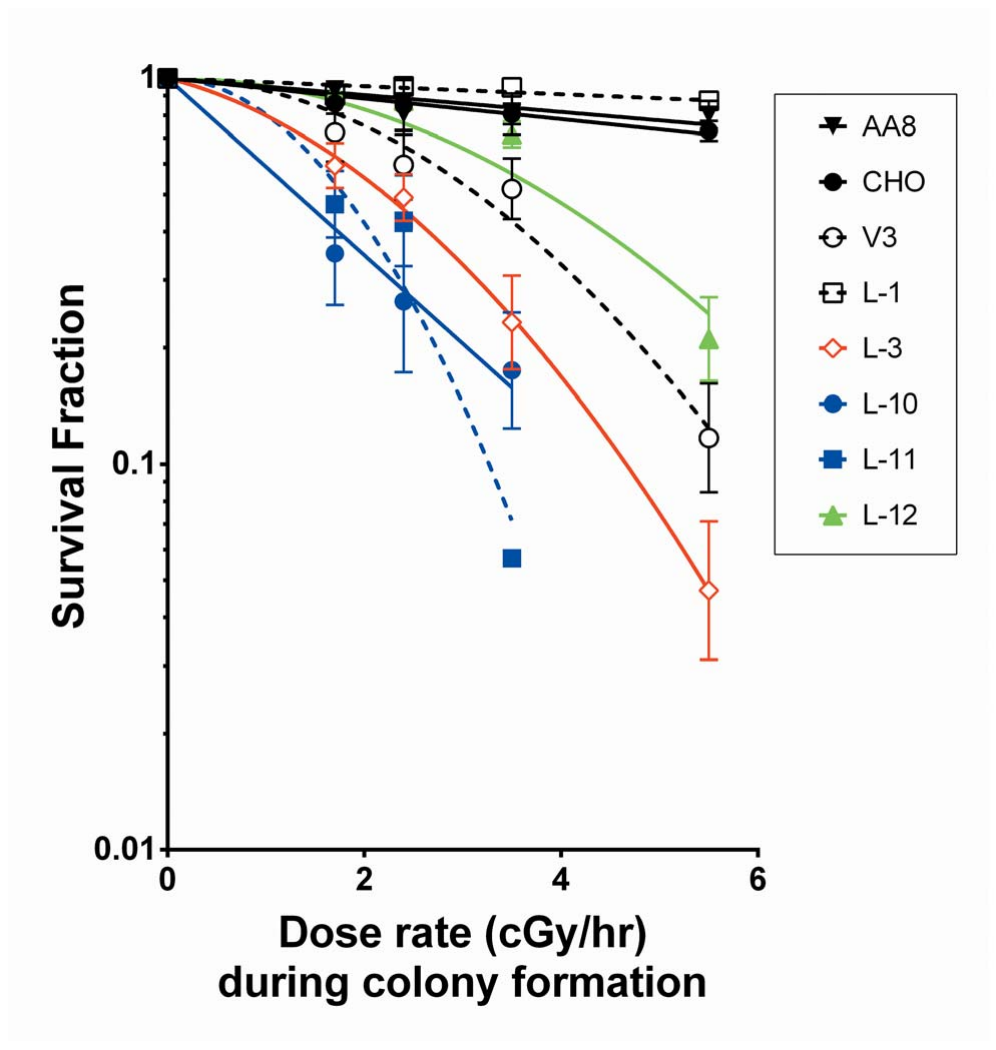
Effect of continuous low-dose-rate irradiation on colony formation. Asynchronous cells were continuously irradiated at a variety of low-dose rates for 8 days. A colony with more than 50 cells was scored as a survivor. Means ± SEMs from more than three independent experiments are shown.

In cells proficient in so-called sub-lethal damage repair, there are marked reductions in effect per unit dose occur when radiation was delivered at low dose rates [44], [45]. For dose rates that are sufficiently low, repair proficient wild-type cells are able to form colonies during continuous irradiation at this or lower dose rates. Mutant cells with even relatively minor defects in critical repair systems are less able to cope with the continuous irradiation, and above a critical dose rate have reduced abilities to form colonies during irradiation [17]. Based on these earlier observations, low dose-rate assay was carried out for the DNA-PKcs mutant cell lines used in the present study. In brief, exponentially growing cells were irradiated for 8 days at dose rates ranging from 1.7 to 5.5 cGy/hour. As illustrated in Figure 3, where the ability to form colonies is plotted against the dose rate experienced during the 8 day incubation period for colony formation, none of the three wild-type cell lines evaluated exhibited any significant reduction in colony forming ability during continuous irradiation at any of the dose-rates tested relative to unirradiated cells. The cells that express the L-3, L-10, and L-11 DNA-PKcs mutants, however, appeared to be even more sensitive than the V3 mutant, while the L-12 mutant appeared as intermediate in sensitivity (Fig. 3).

**Figure 4:**
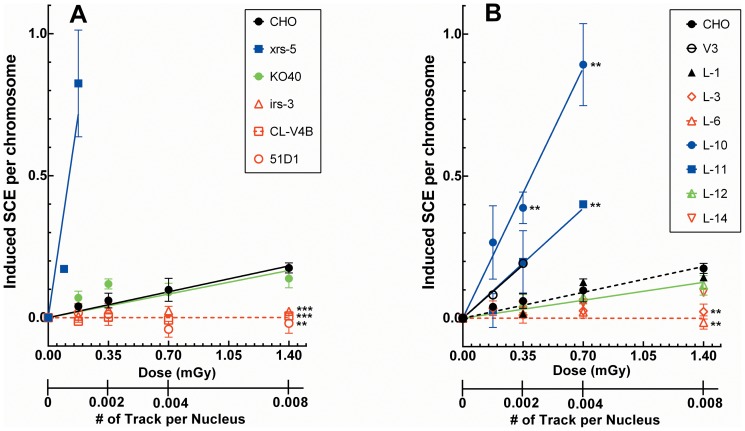
Induction of sister chromatid exchanges with extremely low dose α-irradiation. (A) Wild-type (CHO) and cells deficient in NHEJ (xrs-5), HRR (irs-3, CL-V48, 51D1), and FA (KO40) pathways and (B) V3 cells complemented with wild-type DNA-PKcs (L-1) or with mutants defective in T2609 cluster (L-3/6/14), S2056 cluster (L-12), or PI3K domain (L-10/11) were irradiated with extremely low doses of α-particles. SCEs were scored and normalized to the basal level of SCEs. Means ± SEMs from more than three independent experiments are given. Statistical analyses on the induced frequencies of SCE relative to CHO cells were performed using *t*-test. **, p<0.01; ***, p<0.001.

The baseline dose-response for SCEs induced by α-particles in wild-type cells is shown from 0 to 1.4 mGy for CHO, and L-1 (V3 corrected) cells in Figure 4. At the extreme low end of the sensitivity for SCE induction HRR-deficient CL-V4B (Rad51C), irs-3 (Rad51C), and 51D1(Rad51D) cells (Fig. 4A) as well as cells expressing DNA-PKcs with mutations in the T2609 cluster region (L-3/L-6 cells) (Fig. 4B) showed little or no SCE induction after α-particle irradiation. At the other extreme, the radiation sensitive xrs-5, V3, and L-10/L-11 (PI3K domain mutant or kinase dead) cells displayed large increases in SCE after 0.35 and 0.7 mGy α-particle irradiation (Figs. 4A and 4B). This contrasts with the very low or absent induction of SCEs observed in DNA-PKcs mutants L-3 and L-6 cells. Although FA-deficient KO40 cells displayed only intermediate radiosensitivity (P. Wilson, unpublished data), a moderate increase in SCE was observed in KO40 cells similar to the dose response for wild-type cells over the dose range tested (Fig. 4A) and this also appeared to be the case for L-12 (multiple S2056 cluster) and L-14 cells (see table 1) (Fig. 4B). Thus, greatly increased induced SCE frequencies were found in extreme radiosensitive xrs-5 and some of the more radiosensitive DNA-PKcs mutant cell strains including V3, L-10, and L-11, but in other instances the induced SCE frequencies were either similar to that for wild-type cells or in some cases no SCEs were induced at all. (see Figure 4 and Table 2 for induced frequencies at 0.7 mGy or, on average, 1 track traversal per 250 cell nuclei).

## Discussion

We previously reported that V3 cells that express DNA-PKcs with substitution mutations in involving various regions of the protein displayed differential radiosensitivities when irradiated during the G_1_ phase of the cell cycle [12]. V3-derivative cells expressing DNA-PKcs mutants at the T2609 cluster (L-2 and L-3) and the PI3K domain (L-10 and L-11) were extremely radiosensitive for cell killing when irradiated in the G_1_ phase. In the current study, we observed the general trend that these DNA-PKcs mutant cells show increased radioresistance relative to their G1 responses when irradiated after cells had progressed into late S/G_2_ phase of cell cycle (Fig. 1A–D). Ku70/80-deficient xrs-5 cell survival, however, was independent of the cell cycle occupied at the time of irradiation. In this, the Ku70/80-deficient cells are similar to ATM-deficient cells, which are extremely sensitive to radiation, display no cell-cycle dependence of radiosensitivity, are deficient in SLD and PLD repair, and have a high frequency of radiation-induced chromosomal aberrations relative to wild-type cells [18]–[25], [46].

The lack of a cell-cycle effect in ATM-deficient cells is likely due to the fact that ATM is involved in repair of DSB in both NHEJ and HRR pathways [47], [48]. On the other hand, Ku protein is essential for DSB recognition and DNA-PKcs recruitment through only the NHEJ mechanism [1]. The lack of cell cycle effect on cell survival in xrs-5 cells could be explained by the fact that Ku protects DSB ends from non-specific processing [49], and thereby reduces the frequency of chromosomal exchange aberrations that might otherwise develop. The lack of Ku then might further reduce the proportion of break-pairs that are able to form exchanges, while at the same time resulting in more chromosomal breaks that fail to rejoin altogether resulting in deletion type aberrations. Such a blocking effect on DSB mis-rejoining may occur throughout all cell cycle phases as Ku exhibits similar kinetics for DSB rejoining regardless cell cycle status [50]. As we report here and previously Ku-deficient xrs-5 cells show dramatically increased induction of chromatid breaks but virtually no exchange aberrations when the cells are irradiated in late S/G_2_ phase of cell cycle [41]. Interestingly, large increases in both chromosome breaks and exchanges were seen per unit dose in irs-5 cells when irradiated in G_1_ (figure 2A and 2B). Additionally, Ku possesses 5′deoxyribose-5-phosphate (5′-dRP)/AP lyase activity that results in excision of abasic sites near DSBs *in vitro* [51], although it is not clear whether this activity contributes to cell cycle effects or aberration formation.

Low linear energy transfer (LET) irradiation induces base damage, single-strand breaks (SSBs), DSBs, and cross-links immediately after irradiation [41], [45]. SSBs are rejoined in wild-type cells with a half-time of approximately 10 minutes. DSBs are more slowly rejoined with a half time of 1 to 2 hours [15], [52], [53]. Several reports have indicated that a two-component DSB rejoining system operates with a fast component (t_1/2_ ∼15 minutes) and slow component (t_1/2_ ∼1 to 2 hours) [15], [52]. Mutations leading to defects in dealing with dsbs strongly argue that prompt (or immediate) production of these dsbs by ionizing irradiation are the most important DNA lesions in cell killing, mutation, and tumor promotion for carcinogenesis by irradiation [15], [45], [54].

A close correlation between cell killing and the induction of chromosomal aberrations has also been reported in a number of investigations [46], [52], [55]–[61]. Our results agree with this conclusion. Cells with mutations in components of the NHEJ system were hypersensitive with respect to cell killing and chromosomal aberration induction by γ radiation. The degree of hypersensitivity varied depending on the nature of the mutation, but increases in numbers of chromosomal aberrations were correlated with increases in cell death in each of the cell lines.

In addition to the correlation between cell killing and chromosomal aberration induction, in most, although not all, radiosensitive mutant cells we observed high frequencies of chromosome-type aberrations after G_1_ irradiation and also a very high frequency of chromatid-types aberrations. In wild-type cells, most aberrations were of chromosome types when cells were irradiated in G_1_. This general observation has been reported for many radiosensitive DNA-PKcs mutant cells as well as for lymphocyte and fibroblast cells from ataxia-telangiectasia patients who have mutations in ATM [20], [22], [23], [46], [54], [62]–[67]. Because virtually all base damaging agents that do not produce prompt DNA DSBs result in the production of only chromatid-type aberrations after treatment of G_1_-phase cells, this suggests that ionizing radiation-sensitive mutant cells (e.g. NHEJ and ATM mutations) show both chromatid- and chromosome-type aberrations after G_1_ irradiation because of a concomitant or partially overlapping deficiency or competitive inhibition between DSB and SSB rejoining systems. Late S/G_2_-phase Ku80-deficient xrs-5 cells irradiated with doses of 25 and 50 cGy showed extremely high frequencies of total aberrations, and 80–90% were chromatid-type aberrations (Fig. 2). Although xrs-5 cells had an extremely long G_2_ delay as compared with most other cell lines analyzed upon treatment with relatively low dose irradiation, these cells did progress from the G_2_ checkpoint into mitosis [43]. Hashimoto and colleagues reported that the Ku 80-deficient cells apparently do not adequately repair DSBs before moving into mitosis [68]. They suggested that incompletely repaired DSBs result in SSBs that may appear as chromatid-type breaks in mitosis. This may be pertinent to the mechanism of G_2_-chromosomal hypersensitivity originally suggested by Sanford and colleagues [69] and by Scott [70].

Three types of dose response curves for SCE induction were observed when DNA-PKcs mutant cells were irradiated by extremely low-dose α-particle irradiation (Fig. 4). Moderately radiosensitive L-12 (S2056 cluster mutant) cells showed similar (perhaps slightly lower) frequencies of SCEs relative to wild type (CHO and L-1) cells with up to 1.4 mGy α-particle irradiation (Fig. 4B). Only 0.8% of the nuclei were traversed by an α-particle by this dose (i.e., only one cell nucleus in 125 cells was traversed by an α-particle), so most of the surviving cells expressing SCEs occurred in unirradiated bystander cells. There were very few or no SCEs in L-3, L-6, and L-14 cells (T2609 cluster mutant) with up to 1.4 mGy α-particle irradiation (Fig. 4B). This suggests an overlap in the functional operation of the HRR system and the DNA-PKcs T2609 cluster mutant cells of the NHEJ system. The lack of SCE induction with extremely low dose α-particle fluences has been previously reported in CHO cell lines deficient in Rad51 paralogs (Rad51C, xrcc2, xrcc3) as well as another essential HRR protein Brca2 [26], [27], [71]. This was confirmed for other HRR mutant cells (irs-3, CL-V4B, and 51D1), which also lacked the ability to form SCEs in this study (Fig. 4A). We previously reported that mouse DNA-PKcs 3A knock-in mutant cells (identical design to the L-14 cell line used in this study) were defective in both HRR and FA repair pathways [72]. The lack of SCE induction in L-3, L-6, and perhaps L-14 cells suggests that each individual phosphorylatable residue in the T2609 cluster might contribute specifically and distinctively to the functional efficiency of the HRR process.

When cells were irradiated during the G_1_ phase, the extent of cell killing depended on the number of amino acid replacements within the T2609 cluster. We previously reported that L-6 cells with a single residue replaced had near wild-type sensitivity, L-14 with three residues replaced was of intermediate sensitivity, and L-3 cells with six residues replaced was very radiosensitive [12]. The high radiosensitivity of the L-3 mutant was confirmed in the present study. Therefore, each phosphorylation in the T2609 cluster contributes to radiosensitivity after low LET irradiation based on their functionality underlying the NHEJ mechanism, and phosphorylation of these T2609 residues also influences the interaction with the HRR system to allow SCE induction after low dose α-particle irradiation.

In contrast to the lack of SCE induction in L-3, L-6, and L-14 cells (T2609 cluster mutants), a significant increase in SCEs relative to wild-type cells were observed in L-10 and L-11 (PI3K mutant) cells. The L-10 and L-11 mutations led to approximately 3 and 10 times higher α-particle induced SCE frequencies, respectively, than observed in wild-type cells after treatment with 0.7 mGy (i.e., 0.4% of nuclei traversed by an α-particle) (Fig. 4B). Furthermore, relative to wild-type cells, Ku70/80-deficient xrs-5 cells showed enormously increased SCE frequencies at 0.13 or 0.17 mGy of α-particle irradiation, where on average less than 1 cell nucleus per 1000 cells was traversed by an α-particle. The reason for difference in sensitivity of one set of NHEJ mutants relative to the other with respect to SCE induction suggests the possibility that the wild-type NHEJ system has a minor modulating effect on the HRR system which is required for SCE formation after α-particle irradiation [26], [27]. Severely reducing or eliminating the NHEJ function resulted in extreme hypersensitivity to cell killing and chromosomal aberration induction by low LET radiation but also removed any interference with the HRR system required for α-particle-induced SCE. The mutation in DNA-PKcs that affected phosphorylation of five residues in the S2056 cluster had only a modest effect on sensitivity of cells to γ-rays and no effect on HRR-dependent induction of SCE by α-particles.

In summary, the present study investigated the differential radiosensitivity phenotypes of cells expressing different DNA-PKcs mutants with comparison to cell lines defective in NHEJ or HRR components. Our new analyses are critical as we proceed with further mechanistic studies of DNA-PKcs mutations and their implication in carcinogenesis or other diseases. Significant alteration of DNA-PKcs expression has been correlated with cancer progression and resistance to radio- and/or chemotherapy treatment [73], although less emphasis has been put on mutation spectra analysis of DNA-PKcs probably due to the difficulty of analyzing the enormous DNA-PKcs-encoding *PRKDC* gene. Nonetheless, several point mutations in DNA-PKcs have been identified from breast tumor biopsies including a missense mutation that results in a Thr to Pro substitution at residue 2609 [74]. It is highly plausible that this substitution in the T2609 cluster is the driver for mutation accumulation, genome instability, and eventually carcinogenesis. With the advancement in deep sequencing techniques, it is foreseeable that DNA-PKcs mutations will be identified from tumor biopsies or other diseases. Our current analyses provide information necessary to delineate the molecular mechanism of phenotypic differences resulting from mutations in DNA-PKcs. Phenotypes and molecular mechanisms underlying them are not always predictable from genotypes.
